# Evaluation of the Proximity of Singaporean Children’s Dietary Habits to Food-Based Dietary Guidelines

**DOI:** 10.3390/nu11112615

**Published:** 2019-11-01

**Authors:** Iain A Brownlee, Jasmine Low, Naageswari Duriraju, Mavis Chun, Jessica Xiu Yan Ong, Mia Eng Tay, Gilly A Hendrie, Lourdes Santos-Merx

**Affiliations:** 1 Faculty of Health and Life Sciences, Northumbria University, Newcastle-upon-Tyne NE1 8ST, UK; 2 Newcastle Research and Innovation Institute, Devan Nair Building, Singapore 600201, Singapore; jasminelow93@gmail.com (J.L.); naageswariduriraju@gmail.com (N.D.); jessicaxiuyan@gmail.com (J.X.Y.O.); Gilly.Hendrie@csiro.au (G.A.H.); lourdes.santos-merx@dmu.ac.uk (L.S.-M.); 3 CSIRO Nutrition & Health Program, SAHMRI Building, Adelaide SA5000, Australia; mavischunmc@gmail.com; 4 School of Chemical & Life Sciences, Nanyang Polytechnic, Singapore 569830, Singapore; tay_mia_eng@nyp.edu.sg; 5 Institute of Allied Health Sciences Research, De Montfort University, Leicester LE1 9BH, UK

**Keywords:** diet quality, fruits, vegetables, whole grains, dietary pattern, food-based dietary guidelines

## Abstract

Dietary habits in children may not only impact current health status but could also shape future, lifelong dietary choices. Dietary intake data in Singaporean children are limited. The current study aimed to define the overall diet quality of Singaporean children using an existing cross-sectional dataset and to consider how demographic factors (i.e., body mass index (BMI) status, ethnicity, age, and sex) were associated with these scores. Existing, cross-sectional dietary data (*n* = 561 children aged 6–12 years, collected in 2014–2015) from duplicate 24-h recalls were assessed for diet quality using an index based on the Singaporean Health Promotion Board dietary guidelines. Total diet quality scores were calculated from ten different components (frequencies of rice and alternatives, whole grains, fruits, vegetables, meat and alternatives, dairy and alternatives, total fat, saturated fat, sodium intake, and added sugars). Association with demographic factors and BMI category was evaluated by one-way multivariate ANOVA (MANOVA) tests, with Bonferroni post hoc analyses. Median (interquartile range) total diet quality scores were 65.4 (57.1–73.0). Median scores for whole grains (0.0, 0.0–33.4), fruits (24.1, 0.0–65.3), vegetables (36.5, 10.4-89.8), and sodium (58.4, 0.0–100.0) intake were frequently sub-optimal. Children of Malay ethnic origin had statistically lower total diet quality scores ((55.3, 47.5–60.3) vs. other ethnic groups (combined median 65.4 (57.1, 73.0); *p* < 0.001). These findings highlight the need for continuing efforts to improve dietary intake in young Singaporeans and for longitudinal dietary monitoring in this group.

## 1. Introduction

Habitual dietary intake in children is strongly associated with current health status. Beyond this, imprudent eating habits that develop at an early age may continue throughout the life course and are likely to be major determinants of subsequent risk of morbidity [1,2,3,4]. Food-based dietary guidelines were developed in many parts of the world by public health agencies to support the population to make prudent dietary choices. Such dietary templates would be expected to provide adequate nutrients and be associated with a lower risk of non-communicable diseases while also considering cultural norms and food availability [5]. Approaches to evaluate the proximity of an individual’s dietary habits to prudent dietary practices (“diet quality”) were previously described [6]. Food-based dietary guidelines are frequently used to define the ideal parameters (e.g., of food group frequency and estimated nutrient intake) that denote the highest attainable diet quality score within such approaches [6].

Singapore is a highly developed city state, which is currently experiencing an increase in the prevalence of non-communicable diseases [7] and which saw a rapid shift in food supply necessary to support its population [8]. Singapore is a multicultural society, with the largest three ethnic background groups being Chinese, Malay, and Indian [7]. Food-based dietary guidelines are available for different age groups in Singapore [9], and the adherence to food-based dietary guidelines in women of child-bearing age was previously described [10]. However, data on dietary habits in Singaporean children are limited [11], despite increasing rates of high excess body weight from 1992 to 2010. Over 10% of Singaporean children are now believed to be obese [12]. This study, therefore, aimed to use an existing nationally representative dataset on dietary intake in Singaporean children aged 6–12 years [13] to assess overall diet quality in this age group in relation to food- and nutrient-based dietary guidelines. The associations between available demographic factors and BMI category and total diet quality score were also examined to consider whether specific groups may more frequently have poorer diets and, therefore, be key targets of public health-based interventions.

## 2. Methods

Full details of the data collection approach were previously published [13]. Briefly, ethical approval was granted by the Newcastle University Science, Agriculture, and Engineering Ethics Committee on 24 January 2014 (code 14-BRO-053). Dietary data were collected twice (once for a weekday and once for a weekend day) from 561 participants (*n* = 295 females) and their parents/caregivers by home visits using the multi-pass, 24-h dietary recall method. Average daily food frequency and nutrient intake were calculated as weighted means based on weekday and weekend day data. Trained researchers collected height and weight measurements using standardized protocols with tape measures and digital weighing scales (Body Fat Analyzer HBF 362, Omron Healthcare Asia, Singapore) respectively. Questionnaires were used to collect demographic (age, sex, ethnicity) and physical activity data (the International Physical Activity Questionnaire) during the home visits [13]. The current study represented a subsequent analysis of the anonymized dataset. Nutrient data were originally calculated using dietary software (WinDiets^TM^, Robert Gordon University, Aberdeen, UK), with additional food-group coding carried out in Microsoft Excel. The original dataset did not include estimated added sugar intake. These values were calculated using a previously described, systematic method based on approaches to define the added sugar content of a range of foods available in Australia [14].

The food-based dietary guidelines of the Singapore Health Promotion Board [9] were used to evaluate diet quality in an approach similar to the Healthy Eating Index developed in the United States [15]. Ten different dietary components were considered within this scoring system: frequency of intake of rice and alternatives, whole grains (which included all wholegrain foods as previously defined [13]), fruits, vegetables, meats and alternatives (e.g. fish, tofu, and other soy-based products), dairy and alternatives, sodium, and percentage energy consumed as total fat (recommended as less than 30% of total energy), saturated fat (<10% total energy), and added sugars (<10% total energy). In order to correct for energy intake, the ideal intake of each component was expressed per recommended 1000 kcal. In Singapore, the recommended dietary intake of energy differs by age, sex, and physical activity levels for children, and recommendations for intake of food groups or nutrients tended to be different for six-year-olds compared to 7–12-year-olds [16]. The recommended intake per 1000 kcal of each food group/nutrient was subsequently compared to observed intake per 1000 kcal. Each of the 10 components was equally weighted to calculate an overall score (out of a possible 100, where a higher score reflects greater compliance). Further details of the criteria used to define these scores are available online in the Supplementary Table S1. A final overall diet quality score was subsequently calculated with equal weighting of all 10 dietary components. Body weight status was defined using national BMI (body mass index) *z* cut-off values for Singaporean children [17,18].

### Statistical Analysis

Data were initially evaluated for normality using SPSS version 25.0 (IBM SPSS Inc., Chicago, IL, USA). These tests suggested that total diet quality scores followed a normal distribution while individual components did not and, thus, they were log-transformed. All data were subsequently analyzed for correlation with demographic factors (age, sex, and ethnicity) and BMI category by one-way multivariate ANOVA (MANOVA) tests, with Bonferroni post hoc analyses. A *p*-value of <0.05 was considered statistically significant. For consistency, all data are presented as medians (25th percentile, 75th percentile).

## 3. Results

Table 1 summarizes the diet quality scoring across this cross-sectional sample of Singaporean children. Median total diet quality score of the study sample was 65.4 (57.1, 73.0), with the lowest median category scores being whole grains (0.0, 0.0–33.4), fruits (24.1, 0.0–65.3), vegetables (36.5, 10.4–89.8), and sodium intake (58.4, 0.0–100.0). A large proportion of participants scored maximal values in most of the other categories (see Table 1).

Three main ethnic groupings were self-reported (Chinese, Indian, Malay—see Table 1 for further details), with all other ethnic backgrounds compiled as “other”. The total diet quality scores for Malay children were statistically lower (*p* < 0.001, degrees of freedom = 3, with a partial η^2^ of 0.033 suggesting a small effect size) than other ethnic origins, with fruit, vegetables, dairy, sodium, and added sugar scores also tending to be lower (see Table 1 for further details). Participants of Indian ethnic origin appeared to consume meats and alternatives less frequently than other ethnic groups (*p* < 0.05, degrees of freedom = 3, with a partial η^2^ of 0.041 suggesting a small effect size). As the variance of ethnic groups did not appear to be equal (Levene’s test of <0.01 for both total and meat scores), these calculations were repeated using Mann–Whitney tests, comparing the potentially divergent group with all other participants. In both cases, this also highlighted statistical significance with small effect sizes (total scores of Malay versus non-Malay participants of *p* < 0.001 and η^2^ of 0.013 and meat scores for Indian versus non-Indian participants of *p* < 0.001 and η^2^ of 0.012).

Total and component scores for male and female participants appeared similar (*p* > 0.05). While age was not consistently associated with total diet quality scores or most category scores, there was a statistical difference between the rice and alternative scores between six-year-olds and seven-year-olds (*p* = 0.038). BMI categories did not appear to be associated with diet quality scores (*p* > 0.05—see Table 1).

## 4. Discussion

The overall findings of the current study suggest that dietary intakes of total rice and alternatives, meat and alternatives, and dairy and alternatives meet or exceed recommended intake frequency in Singaporean children of this age range (*n* = 321 (57%), 421 (75%), and 337 (60%) participants, respectively, scored maximally within these categories). Estimates of fat (*n* = 298 or 53%), saturated fat (*n* = 430 or 77%), and added sugar (*n* = 231 or 41%) intake also appear to frequently fall within dietary guidelines. However, intakes of whole grains (*n* = 77 or 14%), fruits (*n* = 89 or 16%), and vegetables (*n* = 120 or 21%) rarely met recommendations, while sodium intake was often high with only *n* = 150 or 27% of participants consuming less than guideline levels. These findings broadly align with global data, suggesting that low intake of fruits, vegetables, and whole grains and high intake of sodium are common among children [19].

The National Nutrition Survey in Singapore does not currently evaluate dietary intake in individuals below 18 years of age [20]. The previous Student’s Health Survey also included some dietary intake data for those above 13 years of age but was discontinued in 2012 [20]. Our findings qualitatively agree with the most recent data from Singaporean adults and older children, where infrequent consumption intake of fruits, vegetables, and whole grains and high salt consumption were previously noted [20,21,22]. Recent public health initiatives in Singapore also aimed to increase fruit, vegetable, and whole grain intake while limiting intake of salt, sugar, and saturated fat, including approaches to improve dietary habits in the school setting [23,24]. Within the Singaporean education system, children aged six years of age are in the school setting for morning or afternoon sessions each day and, thus, may not consume any main meals (or indeed anything) within the setting. Children aged 7–12 study are likely to consume at least one main meal at school. Both age groups could also consume additional snacks during the school day. While the existing efforts should help improve the quality of food made available to children in school, improving dietary habits with the family (at home or when dining out) is likely to have a greater impact on overall diet quality for children this age, as more meal occasions frequently occur outside of the school setting. Recent home-based dietary intervention studies in children would support this notion [25,26].

Recently published data from the GUSTO prospective cohort study on diet quality in 561 Singaporean infants (aged 12–24 months) [27] highlighted similar trends to our study cross-section. Within this study, Chen et al. (2019) used parent-reported food frequency questionnaires to estimate dietary intake and used a Diet Quality Index scoring system with a maximal value of 65. Despite the difference in approaches to estimating dietary intake and calculating dietary quality, median scores from the previous study (68% of maximal scores) were similar to those in the current study (65.4%). Intakes of fruits and vegetables were frequently below recommended amounts with infrequent consumption of foods high in sugar also noted within the GUSTO infant cohort. However, the intake frequency of rice, meat, and dairy products and their alternatives seemed to less frequently meet recommended levels compared to the current study’s participants. Estimated frequency of servings of whole grains was not quantified in the GUSTO study, but there appeared to be a higher proportion of whole-grain consumers (67%) than previously described [13] in the dataset used for the current study (38.3%). This could suggest positive longitudinal changes to the inclusion of whole grains in the diet of young Singaporeans over recent years but may also simply represent a difference in dietary data collection between the two studies.

The original dataset appeared to be representative of the wider population of Singaporean children [13]. Sub-analyses suggest that BMI category, age, and sex were not major factors associated with total diet quality scores. While some previous studies in children of similar ages found an association between higher BMI *z* and lower diet quality scores [27,28,29], this finding is not consistent across all studies [30,31]. While the current dataset appeared to be nationally representative in terms of age, sex, and ethnicity [13], the original study design was not powered to carry out such sub-analyses. A relatively small number of individuals were not within the ideal BMI weight category (*n* = 50 underweight and *n* = 82 overweight), thus limiting the potential to note such associations with a high degree of certainty.

Participants of Malay ethnic origin had statistically lower total diet quality scores, which other recent studies also noted in Singaporean children and adults [27,32,33]. Malay children also consumed markedly lower amounts of fruit on average. It must be noted that all ethnic groups had low median scores for the same components (fruits, vegetables, whole grains, and sodium). These dietary components, therefore, appear to represent the most relevant targets to improve at a national level, in both children and adults, thus representing clear and consistent targets for improving dietary intake at a national level. Median (interquartile range) values for added sugar scores in Malay children (6.8 (0.0, 76.6)) appeared markedly lower than other groups (combined median 83.9 (22.4, 100), suggesting there may be a need for a more targeted intervention in relation to this dietary factor. Additional engagement of children, their caregivers, and households representing different ethnic minorities could represent a means to ensure that cultural issues specific to individual demographics/BMI categories and those shared across the population can be considered in designing future national public health interventions. These findings suggest a need to support Malay children in improving food choices. There may be limitations in culturally acceptable or available prudent food options for this ethnic group requiring further consideration.

The current approach to scoring overall dietary quality based on dietary data is based on each component being arbitrarily equally weighted in its contribution toward the final score. The Dietary Guideline Index used in Australia was developed to be partially weighted based on national dietary practice, with a larger component of the scoring system linked to intake of “discretionary” foods [34]. As such, small differences in total diet quality score values for individuals or average scores between groups may not effectively represent the biological importance of the dietary differences. Larger differences may be more meaningful but alone may not represent similar changes to dietary habits. Our data suggest commonality in overall dietary habits amongst this cross-sectional sample of children. As this approach weighted dietary intake based on total observed energy intake, this helped to limit the chances that individuals who eat large amounts of diverse food groups do not end up with inappropriately high scores. The chances that an individual can end up with a high score based on low reported energy intake are also limited based on the mutual exclusivity of most scoring components.

Dietary intake for each participant in this study was assessed by two 24-h dietary recalls. The major limitation in this approach is the potential for recall bias which may lead to under-reporting [35]. Such data may also not necessarily be representative of habitual dietary intake, as two days of information represents a very short window of dietary evaluation. Food frequency questionnaires are often used to assess habitual intake but may also over-represent intake of foods perceived to be positive for health with a tendency to overestimate total energy intake [36]. In comparison to food frequency questionnaire data, the current approach is perhaps a more objective indication of recent dietary habits. The multi-pass approach for collection of dietary recall data was suggested to improve the accuracy of dietary intake estimation [37].

The collection of longitudinal national nutrition survey data appears necessary to monitor dietary habits in Singaporean children to steer efforts to maintain lifelong health for the population. Approaches based on dietary recalls or food frequency questionnaires, particularly those using online data collection, may represent feasible methods with the lowest participant and researcher burden [35]. Future studies on dietary habits in Singaporean children could consider over-recruiting smaller demographic or BMI category groups to ensure sub-analyses are appropriately powered.

## 5. Conclusions

While some facets of dietary intake in Singaporean children appear to be positive, low intakes of fruits, vegetables, whole grains, and excess sodium, and added sugar consumption appear to be key areas for improvements toward national healthy-eating guidelines. Engagement with children and households from all demographics could inform future public health messaging.

## Figures and Tables

**Table 1:** Median (interquartile range data) for individual components and total diet quality scores by demographics and BMI category.

	Median (Interquartile Range) Scores																
Component	All	Age (Years)							Sex		Ethnicity				BMI Category		
		6	7	8	9	10	11	12	Female	Male	C	I	M	X	U	A	O
*n*	561	81	108	104	85	80	59	44	295	266	434	56	58	13	50	429	82
Rice and alternatives	100.0 (80.5, 100.0)	100.0 (78.9, 100.0) ^a^	92.5 (71.8, 100.0) ^b^	100.0 (72.3, 100.0) ^b^	100.0 (80.9, 100.0) ^b^	100.0 (80.0, 100.0) ^b^	100.0 (83.6, 100.0) ^a, b^	100.0 (94.7, 100.0) ^a, b^	100.0 (80.9, 100.0)	100.0 (78.2, 100.0)	100.0 (78.2, 100.0)	100.0 (87.8, 100.0)	100.0 (85.6, 100.0)	81.3 (76.6, 100.0)	100.0 (85.9, 100.0)	100.0 (79.0, 100.0)	100.0 (82.7, 100.0)
Whole grains	0.0 (0.0, 33.4)	0.0 (0.0, 33.4)	0.0 (0.0, 41.8)	0.0 (0.0, 38.2)	0.0 (0.0, 24.1)	0.0 (0.0, 8.6)	0.0 (0.0, 70.4)	0.0 (0.0, 10.7)	0.0 (0.0, 37.2)	0.0 (0.0, 32.3)	0.0 (0.0, 42.9) ^a^	0.0 (0.0, 30.8) ^a^	0.0 (0.0, 0.0) ^b^	0.0 (0.0, 65.5) ^a, b^	0.0 (0.0, 20.5)	0.0 (0.0, 37.7)	0.0 (0.0, 39.0)
Fruits	24.1 (0.0, 65.3)	23.9 (0.0, 62.7)	13.4 (0.0, 41.0)	30.0 (0.0, 63.2)	11.9 (0.0, 48.1)	25.3 (0.0, 62.4)	28.0 (0.0, 93.7)	23.7 (0.0, 73.3)	25.8 (0.0, 68.3)	20.7 (0.0, 62.6)	26.0 (0.0, 68.9) ^a^	27.6 (0.0, 93.5) ^a^	0.0 (0.0, 31.7) ^b^	42.3 (18.1, 48.8) ^a^	19.9 (0.0, 50.1)	22.5 (0.0, 66.5)	30.4 (0.0, 65.3)
Vegetables	36.5 (10.4, 89.8)	36.4 (9.9, 89.8)	35.9 (8.7, 90.5)	28.8 (8.1, 94.8)	54.0 (18.1, 100.0)	23.7 (7.9, 60.3)	33.5 (11.3, 74.5)	34.3 (12.5, 76.3)	39.1 (11.5, 85.7)	34.2 (9.0, 92.6)	41.2 (13.0, 96.8) ^a^	28.3 (7.6, 69.5) ^a^	12.7 (0.0, 43.9) ^b^	35.4 (0.0, 100.0) ^a, b^	37.4 (9.0, 65.7) ^a, b^	38.1 (11.7, 95.7) ^a^	26.5 (4.1, 69.4) ^b^
Meat and alternatives	100.0 (100.0, 100.0)	100.0 (96.6, 100.0)	100.0 (82.8, 100.0)	100.0 (95.3, 100.0)	100.0 (86.6, 100.0)	100.0 (100.0, 100.0)	100.0 (100.0, 100.0)	100.0 (100.0, 100.0)	100.0 (91.0, 100.0)	100.0 (100.0, 100.0)	100.0 (100.0, 100.0) ^a^	84.6 (48.2, 100.0) ^b^	100.0 (100.0, 100.0) ^a^	100.0 (100.0, 100.0 ^a^	100.0 (100.0, 100.0)	100.0 (98.5, 100.0)	100.0 (96.7, 100.0)
Dairy and alternatives	100.0 (62.3, 100.0)	100.0 (62.3, 100)	100.0 (98.7, 100.0)	100.0 (85.6, 100.0)	100.0 (100.0, 100.0)	100.0 (66.8, 100.0)	89.8 (55.4, 100.0)	100.0 (71.9, 100.0)	100.0 (69.7, 100.0)	100.0 (60.1, 100.0)	100.0 (68.5, 100.0) ^a^	100.0 (83.9, 100.0) ^a^	77.9 (36.9, 100.0) ^b^	100.0 (56.1, 100.0) ^a, b^	95.5 (27.2, 100.0)	100.0 (71.2, 100.0)	100.0 (47.7, 100.0)
Sodium	58.4 (0.0, 100.0)	61.5 (6.3, (100.0)	80.2 (8.8, (100.0)	68.0 (19.5, (100.0)	58.8 (13.8, (100.0)	42.5 (0.0, (100.0)	47.2 (0.0, (94.5)	23.5 (0.0, (62.7)	48.2 (0.0, 96.6)	67.7 (8.5, 100.0)	58.7 (2.8, 100.0)	71.1 (5.1, 100.0)	42.0 (0.0, 92.0)	86.3 (30.3, 94.7)	40.5 (0.0, 76.5)	60.9 (3.4, 100.0)	57.6 (0.0, 100.0)
Fat	100.0 (64.6, 100.0)	100.0 (65.9, 100.0)	96.6 (59.8, 100.0)	100.0 (76.5, 100.0)	100.0 (71.1, 100.0)	100.0 (61.6, 100.0)	88.9 (45.6, 100.0)	100.0 (61.5, 100.0)	100.0 (67.1, 100.0)	97.7 (53.0, 100.0)	100.0 (62.0, 100.0)	100.0 (81.6, 100.0)	99.0 (60.5, 100.0)	100.0 (74.6, 100.0)	100.0 (75.2, 100.0)	100.0 (61.9, 100.0)	100.0 (66.9, 100.0)
Saturated fat	100.0 (100.0, 100.0)	100.0 (100.0, 100.0)	100.0 (99.1, 100.0)	100.0 (100.0, 100.0)	100.0 (99.3, 100.0)	100.0 (97.6, 100.0)	100.0 (93.9, 100.0)	100.0 (100.0, 100.0)	100.0 (100.0, 100.0)	100.0 (100.0, 100.0)	100.0 (100.0, 100.0)	100.0 (100.0, 100.0)	100.0 (92.5, 100.0)	100.0 (95.7, 100.0)	100.0 (99.9, 100.0)	100.0 (100.0, 100.0)	100.0 (98.0, 100.0)
Added sugar	76.9 (11.9, 100.0)	78.0 (9.8, 100.0)	78.1 (13.7, 100.0)	90.0 (18.9, 100.0)	78.9 (7.2, 100.0)	86.6 (26.2, 100.0)	81.6 (43.8, 100.0)	69.2 (20.8, 100.0)	76.7 (5.8, 100.0)	100.0 (83.7, 100.0)	84.9 (27.2, 100.0) ^a^	74.6 (0.0, 100.0) ^a^	6.8 (0.0, 76.6) ^b^	74.4 (0.0, 100.0) ^a, b^	73.7 (6.9, 100.0)	69.3 (9.6, 100.0)	94.5 (58.4, 100.0)
Total	65.4 (57.1, 73.0)	65.5 (57.8, 73.3)	66.4 (58.4, 75.8)	66.4 (58.4, 75.8)	67.9 (59.1, 74.5)	63.5, (58.2, 68.9)	65.3 (53.3, 75.0)	65.1 (57.0, 68.8)	65.7 (58.4, 100.0)	68.0 (59.3, 75.1)	67.0 (59.6, 74.7) ^a^	65.4 (60.7, 68.3) ^a^	55.1 (47.7, 60.6) ^b^	64.8 (58.9, 76.2) ^a^	60.8 (55.8, 68.3)	65.8 (57.8, 74.2)	65.6 (58.6, 71.6)

C = Chinese, I = Indian, M = Malay, X = other ethnic origin. BMI = body mass index, U = underweight (including severely underweight), A = acceptable weight, O = overweight (including severely overweight) as defined by BMI *z*-scores specific to age and sex [17,18]. Sub-groups with different superscripts (^a/b^) for each demographic factor/BMI category in a single row are statistically different (*p* < 0.05) by one-way multivariate ANOVA (MANOVA) with Bonferroni’s post hoc test. Superscripts are omitted where all groups are statistically equal for clarity of presentation.

## Supplementary Materials

The following are available online at https://www.mdpi.com/2072-6643/11/11/2615/s1: Table S1: Values used to derive component and total dietary quality scores in Singaporean children.
